# Can we predict the diffusion "sweet-spot" based on a standard cine?

**DOI:** 10.1186/1532-429X-18-S1-W17

**Published:** 2016-01-27

**Authors:** Andrew D Scott, Pedro Ferreira, Sonia Nielles-Vallespin, Dudley J Pennell, David Firmin

**Affiliations:** 1 Cardiovascular Biomedical Research Unit, The Royal Brompton Hospital, London, United Kingdom; 2 National Heart and Lung Institute, Imperial College, London, United Kingdom; 3 National Heart, Lung and Blood Institute, National Institutes for Health, Bethesda, MD USA

## Background

Cardiac diffusion tensor imaging (cDTI) is often performed using a stimulated echo sequence with monopolar diffusion encoding [1, 2]. While diffusion measured with this sequence is modified by strain [1] the standard strain model predicts that the strain effects can be eliminated by imaging at one of two "sweet-spots" in the cardiac cycle where the effects of strain cancel [3]. However, analysis of strain data to calculate the sweet-spot timings (SST) is typically too time consuming to be performed during an exam. Here we hypothesise that the SST is linearly related to the time to peak strain (PST) and PST will be approximately equal to end-systole time (EST) estimated from a standard cine. If so, EST could be used to easily predict SST.

## Methods

2D spiral cine DENSE data [4] from a mid-ventricular short axis slice in 13 healthy subjects was analysed using the DENSE analysis tool from the University of Virginia [5]. Data were acquired on a Siemens Skyra with either a 3D (navigator gated, 0.10 cycles/mm balanced encoding, n = 5) or a 2D encoded protocol (breath hold, 0.06 cycles/m simple encoding), both 30 ms temporal/3.5 × 3.5 × 8 mm^3^ spatial resolution. Global radial and circumferential strain curves were analysed. The SST were defined as the time points during systolic contraction and diastolic relaxation where the strain was closest to the mean strain over the cardiac cycle. Radial and circumferential PST were averaged. EST was estimated as the timing of the image with minimum left ventricular blood pool from a 25 frame retrospectively gated short axis bSSFP cine in a similar plane to the DENSE images.

## Results

The figure (a and b) shows example radial and circumferential strain curves from one subject and the location of the SSTs. The systolic SST occurs later in the cardiac cycle when predicted using radial strain (p < 0.05) and the diastolic SST occurs earlier in the cardiac cycle using radial strain (p < 10^-5^). Diastolic and systolic SST calculated from either the radial or circumferential strain curves show a good correlation with the PST (figure c and table).

The correlation between SST and EST was poor (figure c and table), due to poor correlation between PST and EST (R^2^ = 0.007).

## Conclusions

The diastolic and systolic SST can be predicted using the PST, but the locations vary depending on whether they are calculated using circumferential or radial strain. Radial strain is the larger component and has a greater influence on cDTI, but circumferential strain is more robust. The SST cannot be predicted from the EST, due to the unexpected result that EST does not relate to PST. Future work should consider whether online feature tracking could be used to predict SST. In the absence of strain information sweet-spot cDTI acquisitions should be performed at the mean time to sweet-spot.

**Table 1:** Results of the linear regressions of sweet-spot times with time to peak strain and end systolic time

	Mean sweet spot time ± SD (ms)	Slope [95% confidence intervals]	Offset (ms) [95% confidence intervals]	R-squared
Using time to peak strain				
Systolic radial	166 ± 29	0.59 [0.29 - 0.89]	-33 [-134 - 69]	0.63
Systolic circumferential	154 ± 22	0.49 [0.30 - 0.68]	-11 [-75 - 53]	0.75
Diastolic radial	488 ± 56	1.33 [0.96 - 1.71]	40 [-88 - 168]	0.85
Diastolic circumferential	512 ± 54	1.29 [0.91 - 1.67]	78 [-50 - 206]	0.84
Using time to peak systole				
Systolic radial	166 ± 29	-0.01 [-0.69 - 0.50]	195 [18 - 372]	0.011
Systolic circumferential	154 ± 22	-0.01 [-0.47 - 0.44]	158 [22 - 294]	0.015
Diastolic radial	488 ± 56	0.22 [-0.93 - 1.36]	424 [80 - 767]	0.00043
Diastolic circumferential	512 ± 54	0.26 [-0.86 - 1.37]	436 [102 - 770]	0.023

Linear regression was performed to a model [Sweet-spot = slope × time + offset] in Matlab. SD - standard deviation.

**Figure 1:**
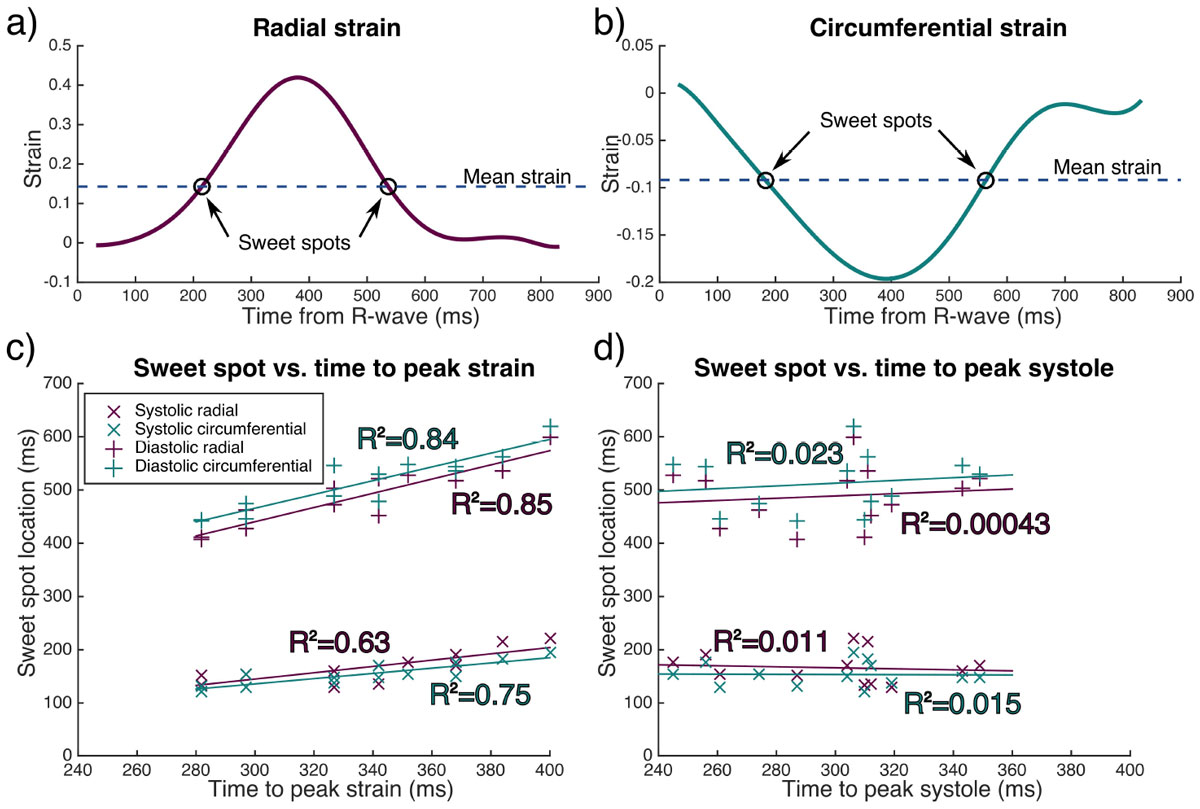
**Radial (a) and circumferential (b) strain-time curves from one example subject showing the location of the sweet-spots**. Scatter plots of the sweet-spot time with time to peak strain (c) and the end-systolic time estimated from a short-axis cine (d) are shown for both the systolic and diastolic sweet spots calculated from both the radial and the circumferential strains.
